# Supplementary material for: Epigenetic signatures of starting and stopping smoking
Source: eBioMedicine. 2018 Oct 30;37:214–20. doi: 10.1016/j.ebiom.2018.10.051 (PMC6286188; doi:10.1016/j.ebiom.2018.10.051)

**Supplementary Figure 1: Proportion of *smoker-enriched* cluster predictions by time since cessation in former smokers, stratified by pack years.**

Proportion of *smoker-enriched* cluster assignments for former smokers is plotted against time since cessation. “Broken stick” regression lines are presented for all former smokers (red solid line, square points), high-dose former smokers (orange dashed line, circular points) and low-dose former smokers (purple dotted line, diamond points), stratified on pack years.


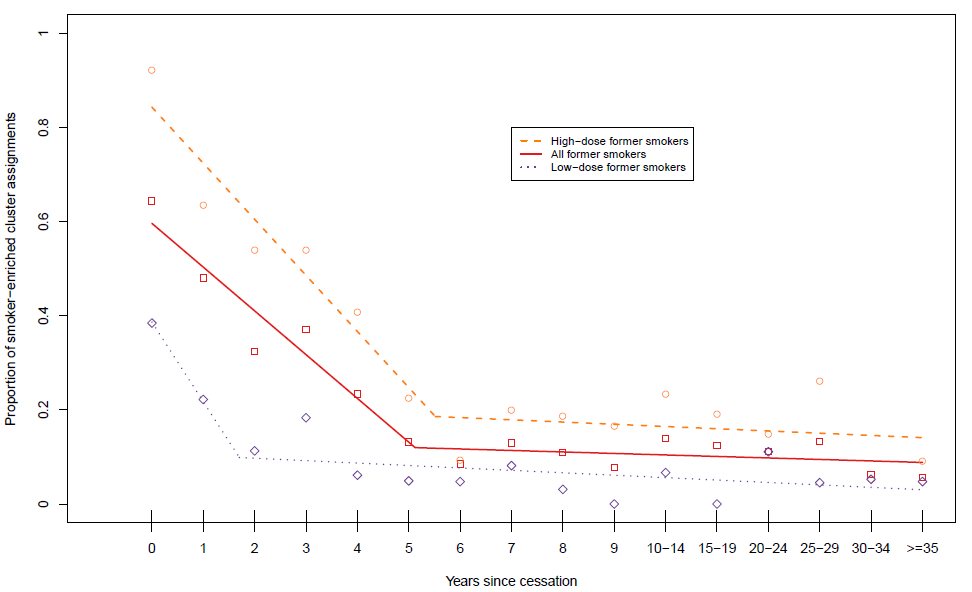


**Supplementary Figure 2: Proportion of *smoker-enriched* cluster predictions by time since cessation in former smokers, stratified by comprehensive smoking index (CSI).**

Proportion of *smoker-enriched* cluster assignments for former smokers is plotted against time since cessation. “Broken stick” regression lines are presented for all former smokers (red solid line, square points), high-CSI former smokers (orange dashed line, circular points) and low-CSI former smokers (purple dotted line, diamond points),
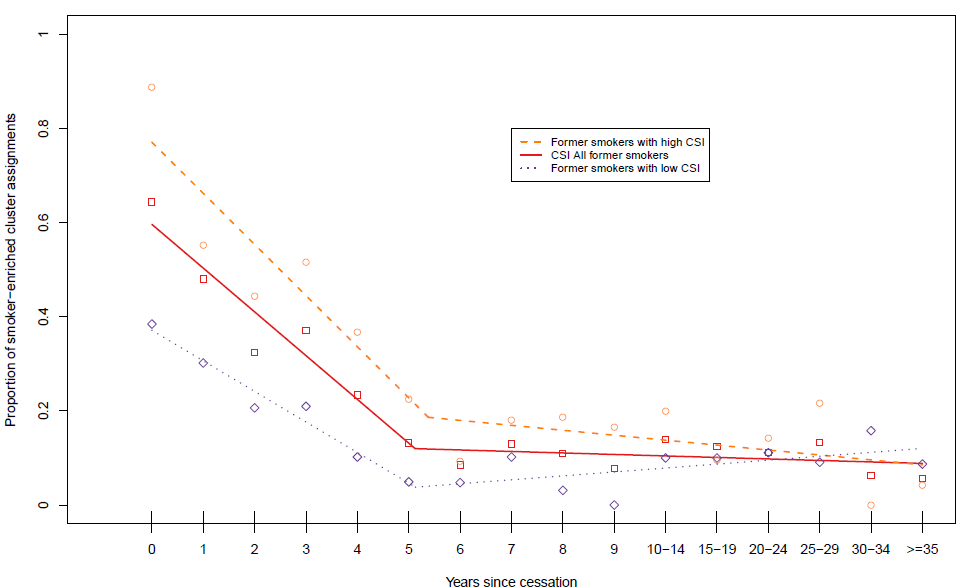


**Supplementary Figure 3: T-statistics for DNA methylation in former versus never smokers at 30 persistent smoking-associated probes.**

T-statistics are presented at individual cessation intervals (A) and cumulative intervals (B) for DNA methylation in former smokers versus never smokers. The blue lines correspond to t-statistics at p = 0.05. The probes that remain significantly differentially methylated at all time points in cumulative former smokers are highlighted in red.


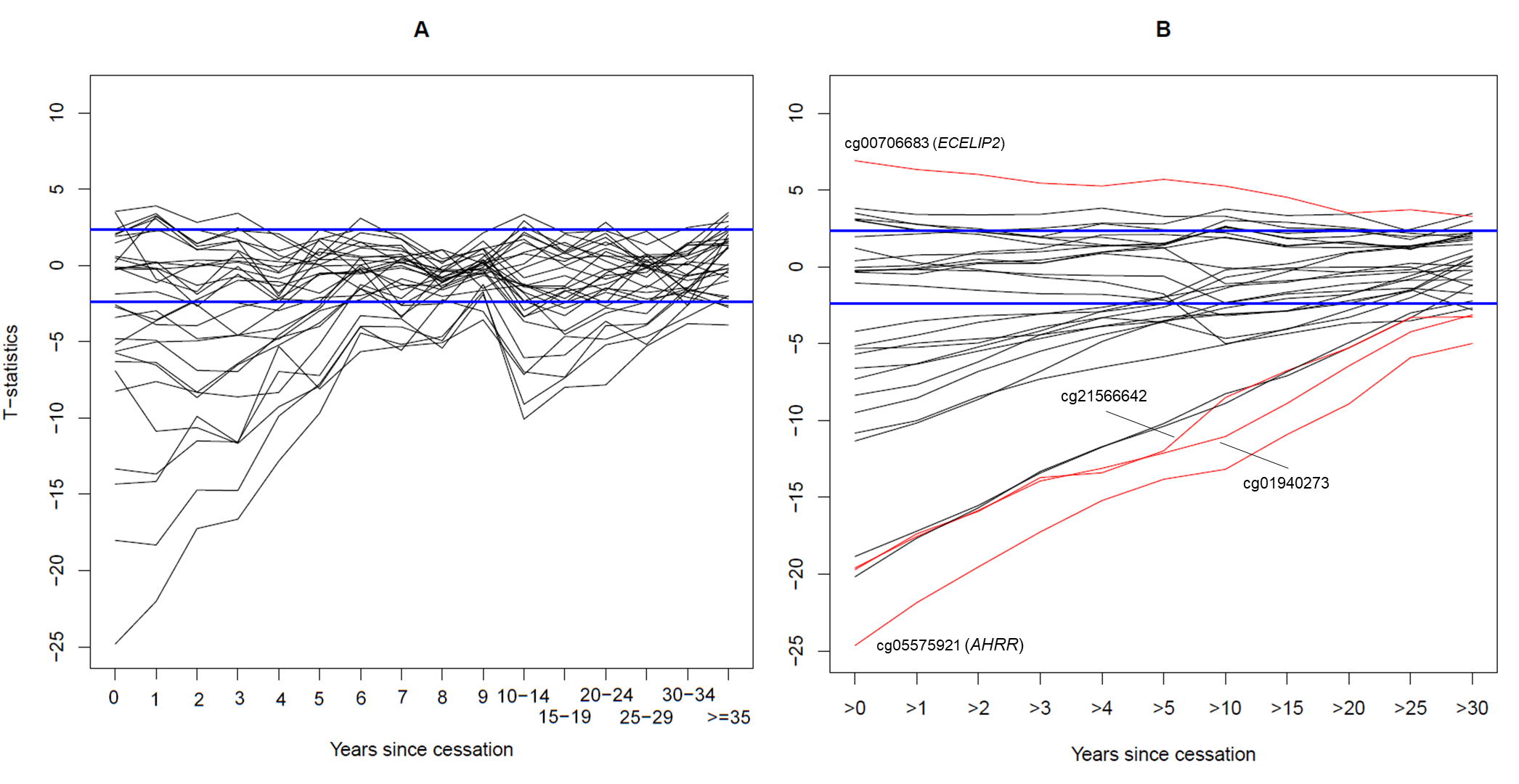


**Supplementary Figure 4: Proportion of current smoker predictions by duration of exposure in current smokers (AUC-based predictions).**

Proportion of *smoker-enriched* cluster assignments for current smokers is plotted against duration of exposure. “Broken stick” regression lines are presented for all current smokers (red solid line, square points), high-dose current smokers (orange dashed line, circular points) and low-dose current smokers (purple dotted line, diamond points).


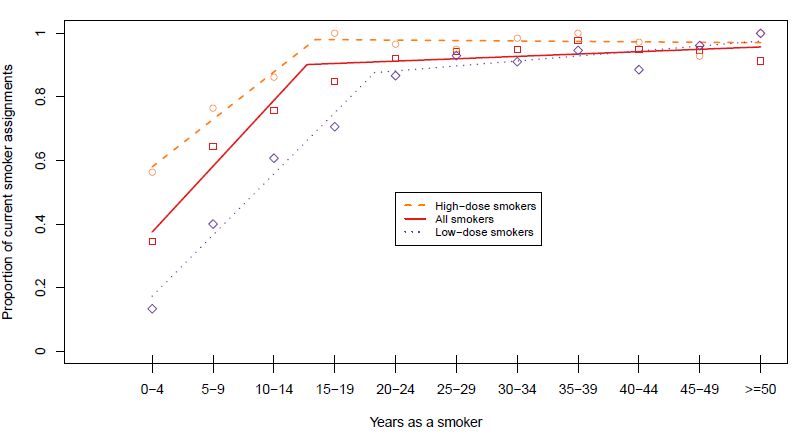


**Supplementary Figure 5: Proportion of current smoker predictions by time since cessation in former smokers (AUC-based predictions).**

Proportion of *smoker-enriched* cluster assignments for former smokers is plotted against time since cessation. “Broken stick” regression lines are presented for all former smokers (red solid line, square points), high-dose former smokers (orange dashed line, circular points) and low-dose former smokers (purple dotted line, diamond points).


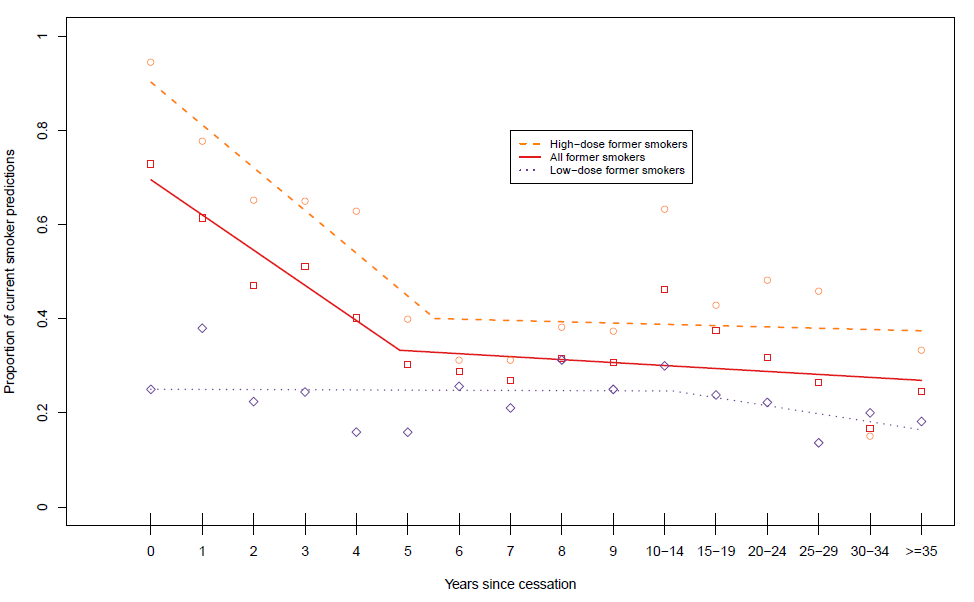


**Supplementary Figure 6: Proportion of current smoker predictions by duration of exposure (90-probe polygenic score-based predictions).**

Proportion of *smoker-enriched* cluster assignments for current smokers is plotted against duration of exposure. “Broken stick” regression lines are presented for all current smokers (red solid line, square points), high-dose current smokers (orange dashed line, circular points) and low-dose current smokers (purple dotted line, diamond points).


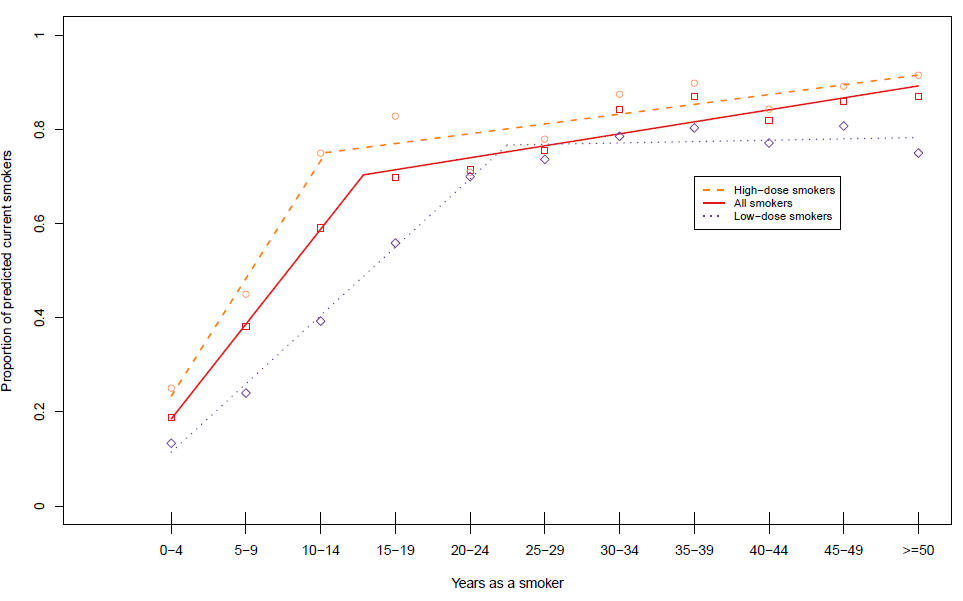


**Supplementary Figure 7: Proportion of current smoker predictions by time since cessation in former smokers (90-probe polygenic score-based predictions).**

Proportion of *smoker-enriched* cluster assignments for former smokers is plotted against time since cessation. “Broken stick” regression lines are presented for all former smokers (red solid line, square points), high-dose former smokers (orange dashed line, circular points) and low-dose former smokers (purple dotted line, diamond points).


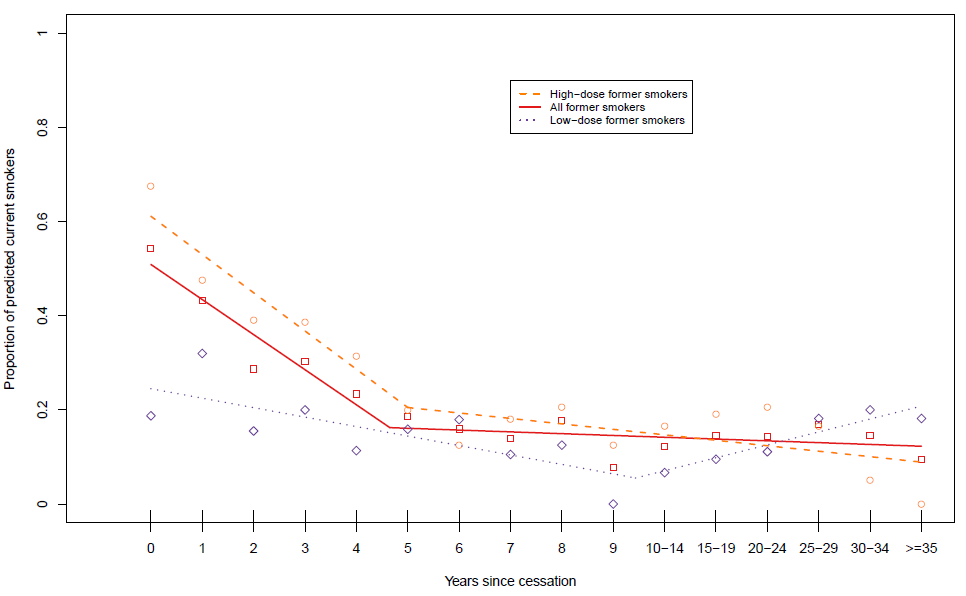


**Supplementary Figure 8: Proportion of current smoker predictions by duration of exposure (17,529-probe polygenic score-based predictions).**

Proportion of *smoker-enriched* cluster assignments for current smokers is plotted against duration of exposure. “Broken stick” regression lines are presented for all current smokers (red solid line, square points), high-dose current smokers (orange dashed line, circular points) and low-dose current smokers (purple dotted line, diamond points).


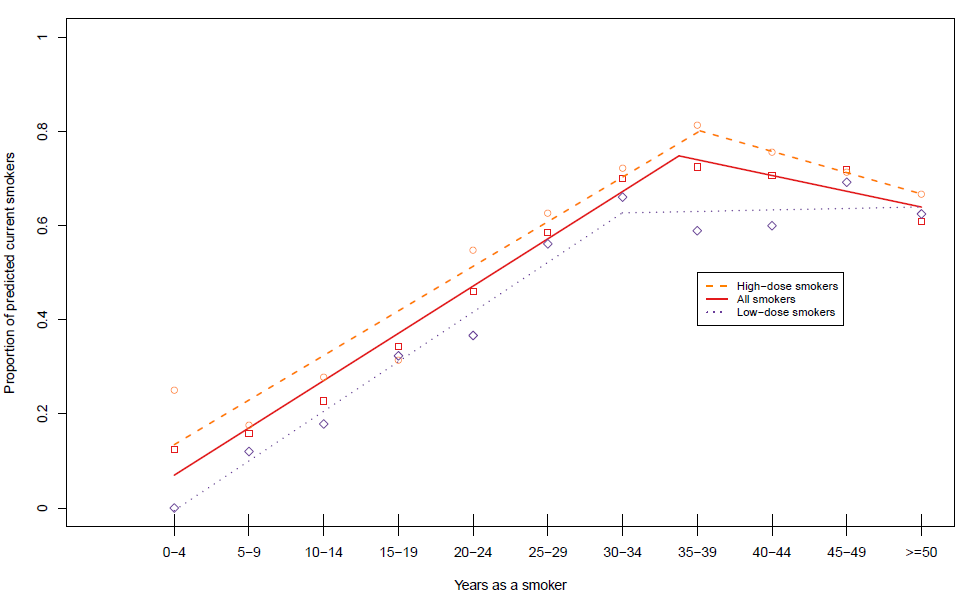


**Supplementary Figure 9: Proportion of current smoker predictions by time since cessation in former smokers (17,529-probe polygenic score-based predictions).** Proportion of *smoker-enriched* cluster assignments for former smokers is plotted against time since cessation. “Broken stick” regression lines are presented for all former smokers (red solid line, square points), high-dose former smokers (orange dashed line, circular points) and low-dose former smokers (purple dotted line, diamond points).


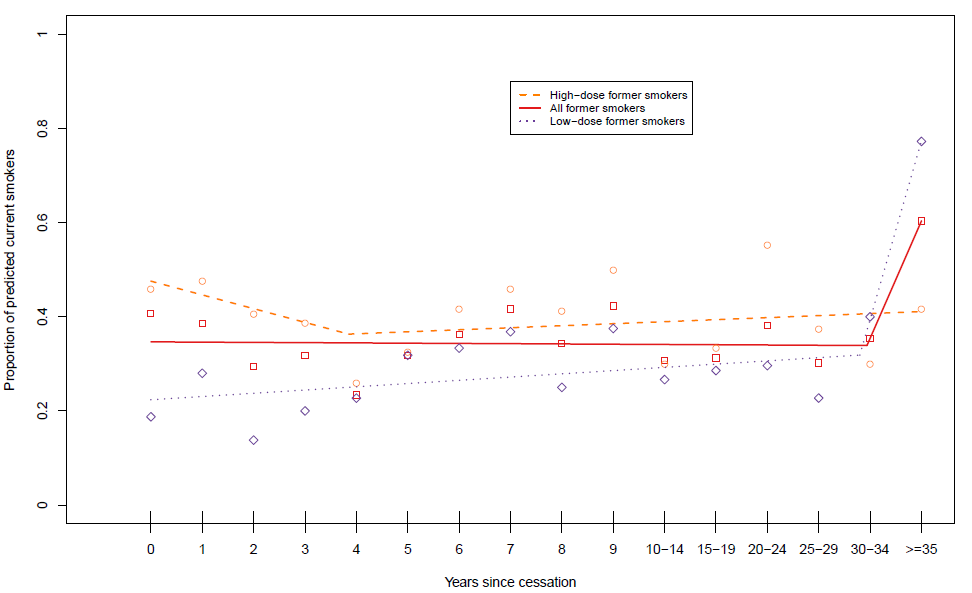


**Supplementary Figure 10: DNA methylation scores versus time since cessation in former smokers.** Box plots are displayed for DNA methylation-based smoking scores in former smokers at 16 cessation time points. The red line corresponds to a “broken stick” regression line for mean smoking score against time since cessation.


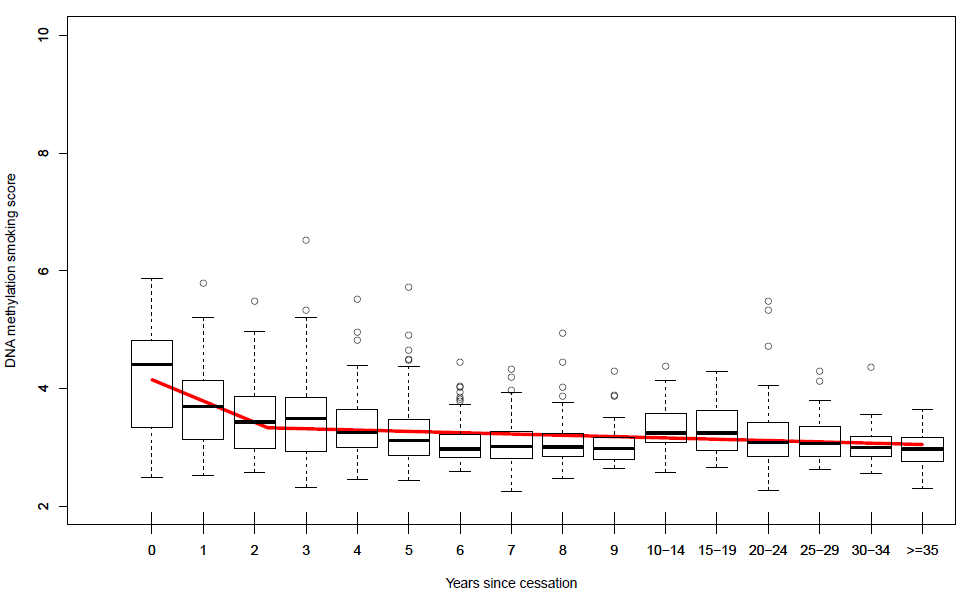

Supplement: Supplementary material 1 [file mmc1.docx]
