# Supplementary material for: Epigenetic signatures of starting and stopping smoking
Source: eBioMedicine. 2018 Oct 30;37:214–20. doi: 10.1016/j.ebiom.2018.10.051 (PMC6286188; doi:10.1016/j.ebiom.2018.10.051)
Supplement: Supplementary material 2 [file mmc2.docx]

**Supplementary Table 1: Proportion of smoker-enriched cluster assignments for current smokers by duration of exposure**

Time points for duration of exposure are shown for current smokers along with sample counts. Proportions of *smoker-enriched* cluster assignments for all samples are presented, along with high-dose and low-dose samples stratified on median cigarettes per day. Sample counts are provided in parentheses. Dose information was not available for all individuals.

| **Duration of exposure** | **N** | **Proportion in *smoker-enriched* cluster (N)** | | |
| --- | --- | --- | --- | --- |
|  |  | ***All samples*** | ***High-dose samples***  ***(≥ median dose)*** | ***Low-dose samples***  ***(< median dose)*** |
| 0-4 years | 32 | 21·9% (32) | 31·3% (16) | 13·3% (15) |
| 5-9 years | 76 | 44·7% (76) | 53·0% (51) | 28·0% (25) |
| 10-14 years | 66 | 63·6% (66) | 75·0% (36) | 44·4% (27) |
| 15-19 years | 73 | 76·7% (73) | 97·1% (35) | 55·9% (34) |
| 20-24 years | 63 | 87·3% (63) | 93·5% (31) | 80·0% (30) |
| 25-29 years | 123 | 89·4% (123) | 89·8% (59) | 91·1% (56) |
| 30-34 years | 140 | 89·3% (140) | 94·4% (72) | 82·1% (56) |
| 35-39 years | 138 | 95·7% (138) | 100% (70) | 89·1% (55) |
| 40-44 years | 116 | 89·7% (116) | 94·3% (70) | 82·9% (35) |
| 45-49 years | 57 | 91·2% (57) | 92·9 (28) | 88·5% (26) |
| ≥ 50 years | 23 | 82·6% (23) | 100% (9) | 88·9% (9) |

**Supplementary Table 2: Proportion of smoker-enriched cluster assignments for former smokers by time since cessation (cigarettes per day stratification).**

Time points for time since cessation are shown for former smokers along with sample counts. Proportions of *smoker-enriched* cluster assignments for all samples are presented, along with high-dose and low-dose samples stratified on median cigarettes per day. Sample counts are provided in parentheses.

| **Time since cessation** | **N** | **Proportion in *smoker-enriched* cluster** | | |
| --- | --- | --- | --- | --- |
|  |  | ***All samples*** | ***High-dose samples***  ***(≥ median dose)*** | ***Low-dose samples***  ***(< median dose)*** |
| < 1 year | 59 | 64·4% (59) | 81·1% (37) | 25·0% (16) |
| 1 year | 127 | 48·0% (127) | 54·0% (63) | 32·0% (50) |
| 2 years | 136 | 32·4% (136) | 46·4% (69) | 15·5% (58) |
| 3 years | 135 | 37·0% (135) | 48·7% (80) | 15·6% (45) |
| 4 years | 107 | 23·4% (107) | 33·3% (54) | 11·4% (44) |
| 5 years | 129 | 13·2% (129) | 18·8% (80) | 4·5% (44) |
| 6 years | 94 | 8·5% (94) | 8·3% (48) | 5·1% (39) |
| 7 years | 108 | 13·0% (108) | 16·4% (61) | 10·5% (38) |
| 8 years | 73 | 11·0% (73) | 17·6% (34) | 3·1% (32) |
| 9 years | 26 | 7·7% (26) | 12·5% (16) | 0% (8) |
| 10-14 years | 65 | 13·8% (65) | 20·0% (30) | 10·0% (30) |
| 15-19 years | 48 | 12·5% (48) | 14·3% (21) | 4·8% (21) |
| 20-24 years | 63 | 11·1% (63) | 17·2% (29) | 7·4% (27) |
| 25-29 years | 53 | 13·2% (53) | 20·8% (24) | 9·1% (22) |
| 30-34 years | 48 | 6·3% (48) | 5·0% (20) | 10·0% (20) |
| ≥ 35 years | 53 | 5·7% (53) | 8·3% (24) | 4·5% (22) |

**Supplementary Table 4: Relationship between cluster assignment and potential confounders in current and never smokers**

Shown are comparisons in bold, along with their corresponding tests and P-values. Chi-squared tests were performed to assess the difference in proportions of males/females, plate processing batches, and passive smokers in each cluster. Due to the small number of former drinkers in the dataset, Fisher’s exact tests were performed to assess the proportions of alcohol consumption groups in each cluster.

|  | **Test** | **P-value** |
| --- | --- | --- |
| **Cluster vs gender (never smokers)** | Chi-squared test | 0·967 |
| **Cluster vs batch (never smokers)** | Chi-squared test | 0·179 |
| **Cluster vs alcohol consumption (never smokers)** | Fisher’s Exact Test | 0·332 |
| **Cluster vs passive smoking (never smokers)** | Chi-squared test | 0·999 |
| **Cluster vs gender (current smokers)** | Chi-squared test | 0·541 |
| **Cluster vs batch (current smokers)** | Chi-squared test | 0·521 |
| **Cluster vs alcohol consumption (current smokers)** | Fisher’s Exact Test | 0·103 |
| **Cluster vs rs1051730 genotype (current smokers)** | Logistic regression | 0·222 |

**Supplementary Table 5: Proportion of smoker-enriched cluster assignments for former smokers by time since cessation (pack years stratification)**

Time points for time since cessation are shown for former smokers along with sample counts. Proportions of *smoker-enriched* cluster assignments for all samples are presented, along with high-dose and low-dose samples stratified on median pack years.

| **Time since cessation** | **N** | **Proportion in *smoker-enriched* cluster** | | |
| --- | --- | --- | --- | --- |
|  |  | ***All samples*** | ***High-dose samples*** | ***Low-dose samples*** |
| < 1 year | 59 | 64·4% | 92·3% | 38·5% |
| 1 year | 127 | 48·0% | 63·6% | 22·2% |
| 2 years | 136 | 32·4% | 54·0% | 11·3% |
| 3 years | 135 | 37·0% | 54·0% | 18·3% |
| 4 years | 107 | 23·4% | 40·8% | 6·1% |
| 5 years | 129 | 13·2% | 22·6% | 4·9% |
| 6 years | 94 | 8·5% | 9·3% | 4·8% |
| 7 years | 108 | 13·0% | 20·0% | 8·2% |
| 8 years | 73 | 11·0% | 18·8% | 3·1% |
| 9 years | 26 | 7·7% | 16·6% | 0% |
| 10-14 years | 65 | 13·8% | 23·3% | 6·7% |
| 15-19 years | 48 | 12·5% | 19·0% | 0% |
| 20-24 years | 63 | 11·1% | 14·8% | 11·1% |
| 25-29 years | 53 | 13·2% | 26·1% | 4·5% |
| 30-34 years | 48 | 6·3% | 5·3% | 5·3% |
| ≥ 35 years | 53 | 5·7% | 9·1% | 4·8% |

**Supplementary Table 8: Predicted current/never smoking status based on 5 probes with AUC > 0.9**

|  | **Predicted Current Smoker** | **Predicted Never Smoker** |
| --- | --- | --- |
| **Current Smoker** | 781 | 136 |
| **Never Smoker** | 33 | 2,489 |

**Supplementary Table 9: Predicted current/never smoking status based on polygenic score from 90 probes:**

|  | **Predicted Current Smoker** | **Predicted Never Smoker** |
| --- | --- | --- |
| **Current Smoker** | 672 | 245 |
| **Never Smoker** | 153 | 2,369 |

**Supplementary Table 10: Predicted current/never smoking status based on polygenic score from 17,529 probes:**

|  | **Predicted Current Smoker** | **Predicted Never Smoker** |
| --- | --- | --- |
| **Current Smoker** | 500 | 417 |
| **Never Smoker** | 547 | 1,975 |
